# Automated MRI protocoling in neuroradiology in the era of large language models

**DOI:** 10.1007/s11547-025-02040-9

**Published:** 2025-07-11

**Authors:** Lara Noelle Reiner, Moudather Chelbi, Leonard Fetscher, Juliane C. Stöckel, Christoph Csapó-Schmidt, Shakhnaz Guseynova, Fares Al Mohamad, Keno Kyrill Bressem, Jawed Nawabi, Eberhard Siebert, Mike P. Wattjes, Michael Scheel, Aymen Meddeb

**Affiliations:** 1 Department of Neuroradiology, Charité—Universitätsmedizin Berlin, Augustenburger Platz 1, 13353 Berlin, Germany; 2 Department of Neuroradiology, Hôpital Maison-Blanche, CHU Reims, Université Reims-Champagne-Ardenne, Reims, France; 3 Berlin Institute of Health at Charité—Universitätsmedizin Berlin, Charitéplatz 1, 10117 Berlin, Germany; 4 Department of Radiology, Technical University Munich, Klinikum Rechts Der Isar, Ismaninger Str. 22, 81675 Munich, Germany; 5 Department or Radiology and Nuclear Medicine, Technical University Munich, German Heart Center Munich, Lazarethstr. 36, 80636 Munich, Germany

**Keywords:** Artificial intelligence, Automation, Decision-support systems, Clinical, Large language models, Magnetic resonance imaging, Natural language processing, Neuroradiology

## Abstract

**Purpose:**

This study investigates the automation of MRI protocoling, a routine task in radiology, using large language models (LLMs), comparing an open-source (LLama 3.1 405B) and a proprietary model (GPT-4o) with and without retrieval-augmented generation (RAG), a method for incorporating domain-specific knowledge.

**Material and Methods:**

This retrospective study included MRI studies conducted between January and December 2023, along with institution-specific protocol assignment guidelines. Clinical questions were extracted, and a neuroradiologist established the gold standard protocol. LLMs were tasked with assigning MRI protocols and contrast medium administration with and without RAG. The results were compared to protocols selected by four radiologists. Token-based symmetric accuracy, the Wilcoxon signed-rank test, and the McNemar test were used for evaluation.

**Results:**

Data from 100 neuroradiology reports (mean age = 54.2 years ± 18.41, women 50%) were included. RAG integration significantly improved accuracy in sequence and contrast media prediction for LLama 3.1 (Sequences: 38% vs. 70%, *P* < .001, Contrast Media: 77% vs. 94%, *P* < .001), and GPT-4o (Sequences: 43% vs. 81%, *P* < .001, Contrast Media: 79% vs. 92%, *P* = .006). GPT-4o outperformed LLama 3.1 in MRI sequence prediction (81% vs. 70%, *P* < .001), with comparable accuracies to the radiologists (81% ± 0.21, *P* = .43). Both models equaled radiologists in predicting contrast media administration (LLama 3.1 RAG: 94% vs. 91% ± 0.2, *P* = .37, GPT-4o RAG: 92% vs. 91% ± 0.24, *P* = .48).

**Conclusion:**

Large language models show great potential as decision-support tools for MRI protocoling, with performance similar to radiologists. RAG enhances the ability of LLMs to provide accurate, institution-specific protocol recommendations.

## Introduction

MRI acquisition protocol assignment is a critical step in the radiology workflow, ensuring adequate imaging for patient care. However, this process is time-consuming, accounting for approximately 6.2% of a radiologist’s work shift [1]. Each institution customizes its protocols based on factors such as the availability of MRI machines, examination time constraints, and the specific needs of other clinical departments, such as radiotherapy or surgery. This lack of standardization poses challenges, particularly for less experienced physicians [2]. Manual protocol selection is not only time-consuming but also prone to errors, with higher error rates observed among trainees [3]. Moreover, the continuous influx of new MRI orders disrupts radiologists' workflow, diverting their attention away from critical image interpretation tasks. To address these challenges, several studies have explored the automation of protocol assignment using artificial intelligence (AI), specifically machine learning techniques [4–10].

The introduction of transformer-based large language models (LLMs) represents a major advancement in automated language processing [11], with their application rapidly expanding within the medical field [12]. In radiology, LLMs have been particularly explored for summarizing and simplifying reports, extracting structured data for research purposes and clinical quality assessment [13, 14], and for translating reports to enhance cross-linguistic patient care [15]. LLM-based predictions of certain radiology protocol categories have been investigated, but studies have largely excluded specific MRI protocols and their varied sequences [16].

Due to their autoregressive nature, LLMs are prone to generating responses that sound convincing but are inaccurate, a phenomenon termed hallucination. This is particularly problematic in the healthcare sector, where hallucination can negatively affect patient health. Retrieval-augmented generation (RAG) is a novel approach that helps mitigate this issue, by providing the LLMs with additional context. Specifically, an external database, composed of custom text data, is queried, and the most relevant information for the task is retrieved—similarly to a search engine—and passed to the model, allowing the generation of context-specific responses [17]. This technique is particularly suitable for medical tasks where the required information is not necessarily available in the models initial training data, and where diagnostic and treatment guidelines are continuously evolving. New insights can be easily added to the database and does not require a retraining of the model, contrary to machine learning models.

The use of RAG in medicine has been investigated in studies spanning from answering disease-specific questions to recalling guideline information and providing guideline-specific recommendations, with promising results [18–22].

This study aims to evaluate the performance of LLMs in assigning MRI protocols based on patients’ clinical questions in neuroradiology. It compares the accuracy of responses between an open-source and a proprietary model and examines whether incorporating RAG improves accuracy within the same model.

## Material and methods

### Population

**Fig. 1:**
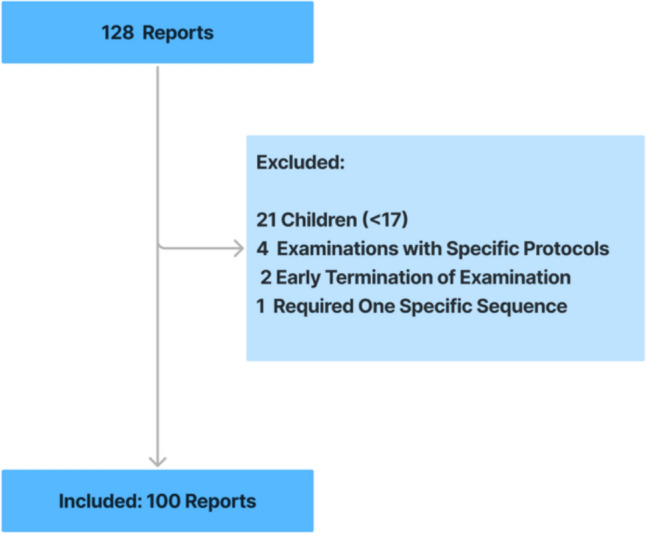
**Report Selection Process**. A fully conducted MRI examination with a neuroradiological question served as inclusion criteria for this study. Exclusion criteria included early examination termination, the use of specific study protocols or requirement of one specific sequence, and patients under the age of 17, as all neuroradiological examinations for this age group follow pediatric MRI protocols

For this retrospective study, MRI examination reports were obtained from the Department of Neuroradiology at a university hospital. The study was approved by the ethics committee of the university (No. EA4/062/20). The need for informed consent was waived due to the retrospective nature of the research. From a total of 13,960 reports from examinations conducted in 2023, a random sample of 128 reports was drawn using the Radiology Information System (RIS) with a fixed random state of 42. This randomized selection minimized selection bias caused by clustering based on disease patterns or the ordering physician in consecutive MRI orders. Selected reports included examinations performed between January 3, 2023, and December 20, 2023. All reports pertained to neuroradiological patients who underwent uninterrupted MRI examinations of the brain or spine, serving as the inclusion criteria for this study. Exclusion criteria and corresponding numbers are detailed in Fig. 1. MRI examinations were conducted with 12 different MRI devices: four with 1.5 T MAGNETOM Aera and eight with 3 T MAGNETOM Skyra (Siemens, Munich, Germany). The administered contrast media were Gadovist® (Bayer, Leverkusen, Germany) or Dotarem® (Guerbet, Villepinte, France). Data collection adhered to the ethical standards of the Declaration of Helsinki, following strict clinical research data protection guidelines. Patient data were anonymized to ensure privacy and confidentiality, following strict clinical research data protection guidelines, and no identifiable patient information was shared with the AI system.

### Data extraction

The RIS dataset was provided as a.csv file containing patients’ demographic data, MRI device names and the corresponding report. Key information, including symptoms, indication, clinical question, and imaging procedure, was extracted from the report using regular expression-based text extraction. The device column was renamed to indicate magnetic field strength due to differences between 1.5 T and 3 T protocols. Data extraction was performed in Python (Vers.: 3.12.3, [23]) using the NumPy and Pandas package (Versions: 1.26.4 and 1.5.3, respectively).

The current protocol guidelines used by the Department of Neuroradiology were provided in a portable document format (PDF) file. To facilitate their use in the RAG system, manual basic formatting adjustments were applied to the document, which contains 63 different MRI protocols.

### Establishment of ground truth

To establish the ground truth, a neuroradiologist with 13 years of experience (J.S.) reviewed all clinical questions and selected the appropriate MRI protocol for each patient based on protocol guidelines.

**Fig. 2:**
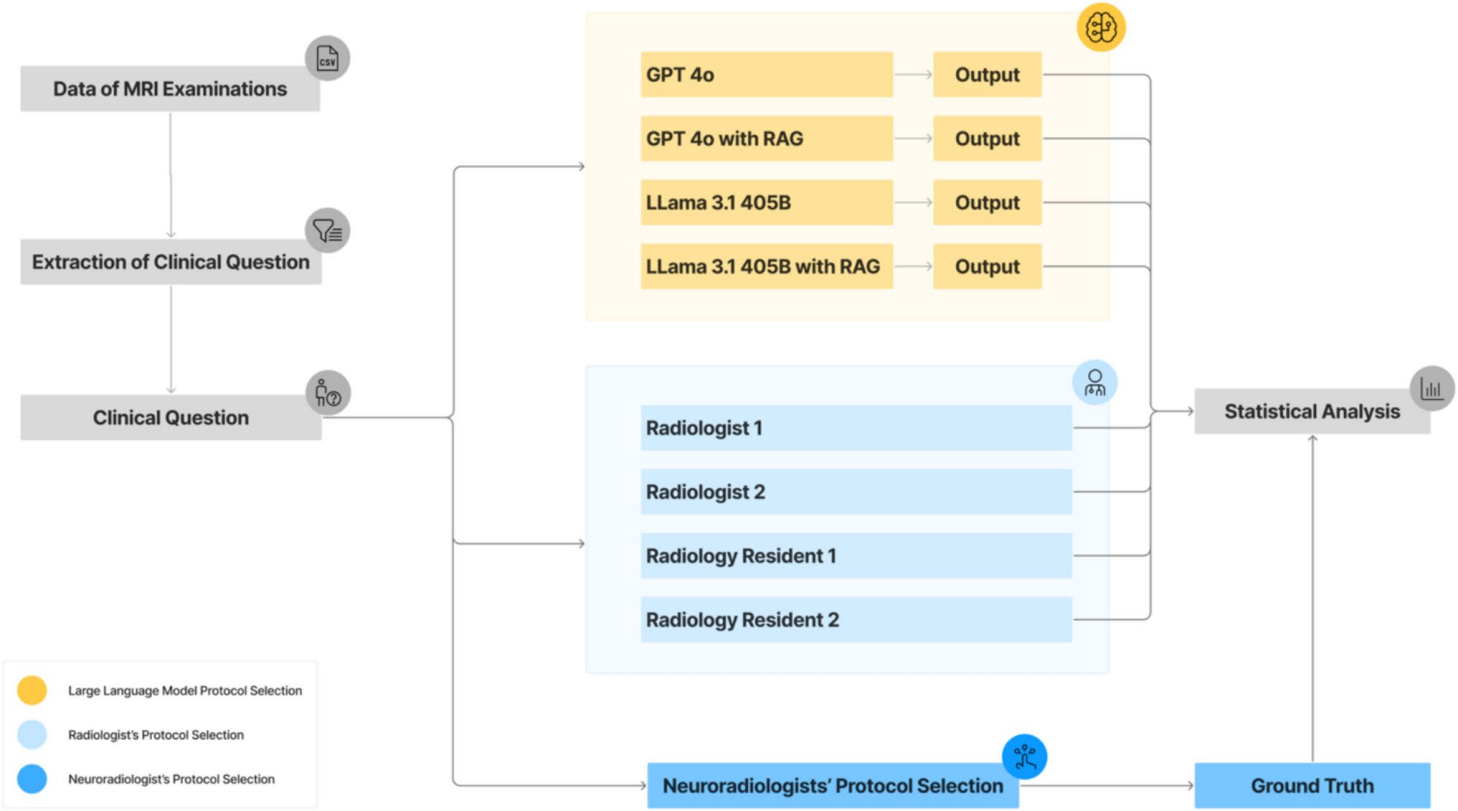
**Study Design**. The clinical question and MRI device data were extracted from MRI reports and provided to an experienced neuroradiologist (J.S., 13 years of experience) for manual protocol selection to establish the ground truth. This information was also used as input for the four tested pipelines. Additionally, four radiologists with varying experience levels performed manual protocol selection for comparison. Statistical analysis was conducted to compare the protocol selections of the large language model and the radiologists against the ground truth

For the comparison of models and radiologists, two board-certified general radiologists with seven years of experience (S.G., J.N.) and two first-year radiology residents (F.A., L.F.) completed the same task. The study design is outlined in Fig. 2.

### Models

We used OpenAI’s GPT-4o (Version gpt-4o-2024–08-06) as a proprietary model and Meta’s LLaMA 3.1 405B as an open-source model, both based on the GPT architecture. GPT-4o, OpenAI’s latest model, released on May 13, 2024, supports up to 128,000 tokens in context, with an estimated parameter size of around 1.8 trillion [24]. Meta’s LLaMA 3.1 405B, released on July 23, 2024, also supports 128,000 tokens and has 405 billion parameters, making it the largest open-source model available at the time [25].

A single embedding model, OpenAI's "text-embedding-ada-002" [24], was used for both RAG pipelines to ensure consistent retrieval results. LLaMa 3.1 405B was run in a non-quantized format on Replicate [26], which provides the necessary computational resources for large open-source models.

### RAG-system

This study distinguishes between two workflows: the non-RAG scenario and the RAG scenario. Both start with the patient's clinical question and aim to predict optimal MRI sequences while also determining the need for contrast medium.

**Fig. 3:**
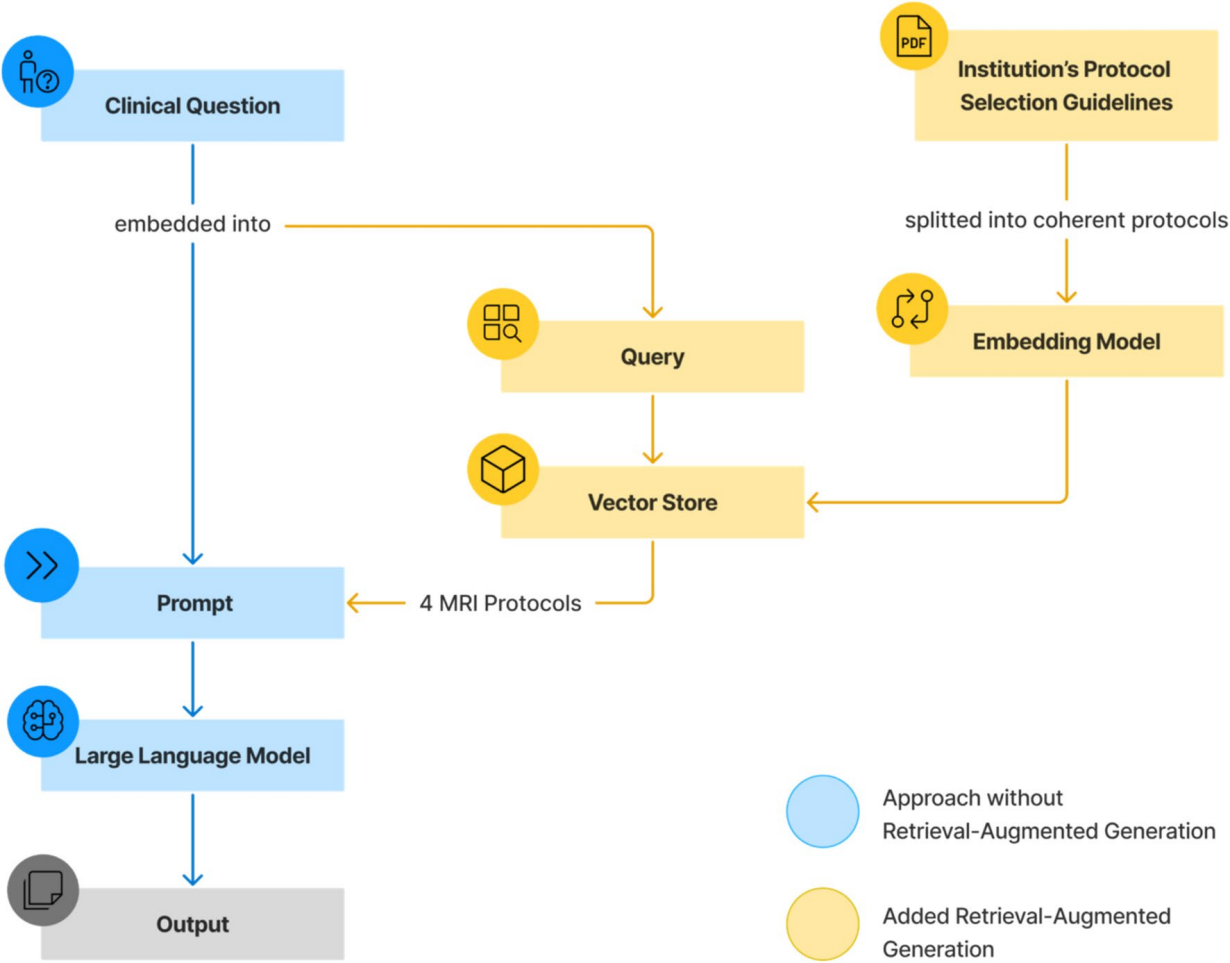
**Combination of Large Language Model and Retrieval-Augmented Generation**. In the non-retrieval-augmented generation (RAG) approach, the clinical question is embedded directly in the prompt, enabling the large language model to predict contrast medium administration and suitable MRI sequences based on our institution’s standard sequences. RAG extends this process by using the clinical question to query the vector store, which is constructed from segmented protocols based on our institution-specific guidelines. Through similarity search, the four most relevant protocols are retrieved and incorporated into the prompt

To set up the RAG-System, the guidelines were first segmented into paragraphs using the recursive character text splitter function by LangChain, with a maximum chunk size of 400 characters and zero overlap, ensuring the separation of the individual MRI protocols. The embedding model converted each paragraph into a continuous, high-dimensional vector representation and stored them in a vector database, thereby capturing semantic relationships and similarities of the text data. The patients’ clinical questions were used to query the data in the vector store. Based on similarity search, the four most relevant MRI protocols were retrieved and subsequently incorporated into the prompt. The constructed setup is depicted in Fig. 3.

In the RAG scenario, the model selects the appropriate protocol from the top four retrieved options and outputs the MRI sequences exactly as listed in the protocol. In the non-RAG scenario where no protocols are specified, the model chooses the MRI sequences from a list that includes all sequences mentioned across protocols, ensuring that the names remain consistent despite non-standardized MRI sequence naming.

To evaluate the model's grasp of medical terminology and reasoning, it was also instructed to explain abbreviations in the clinical question, identify any explicitly mentioned diagnoses, and suggest the three most likely differential diagnoses for the patient.

The prompt was presented to all four models in German, with the temperature parameter set to 0.1 to ensure low variability and high reproducibility. Model access and RAG implementation were handled via an application programming interface (API) in Python (Version: 3.12.3, [23]) using Replicate (Version: 0.32.1), OpenAI (Version: 1.37.1) and LangChain packages (Versions: Community: 0.2.9, Text Splitters: 0.2.2, OpenAI: 0.1.17).

The source code for the methods used in this study is publicly accessible on GitHub [27].

### Statistical analysis

The accuracy of MRI sequence predictions was evaluated using symmetric token-based accuracy. Both the ground truth (GT) sequences and the model’s predictions are split into individual tokens, each representing one MRI sequence (e.g., Axial T1). The metric conducts a bidirectional comparison between the ground truth and the prediction, treating all MRI sequences equally while disregarding their order. With this approach, both missing and superfluous MRI sequences negatively affect accuracy.

Bootstrapping, as a statistical resampling method, was employed to estimate the 95% confidence interval for the accuracy measurements. This method repeatedly samples with replacement from the original output data to approximate the metric’s distribution, allowing accurate confidence interval estimation without excessive, resource-consuming output generation. To assess the agreement among raters, while accounting for the possibility of correct predictions due to random guessing, Cohen’s kappa was calculated for the radiologists’ protocol selection.

For statistical comparison of the LLM outputs and radiologists’ protocol selection, the Wilcoxon signed-rank test was used for MRI sequences, and McNemar test was employed for contrast medium administration, with *P* < 0.05 considered indicative for statistical significance. The mean accuracy and standard deviation were calculated for the radiologists’ results to enable a singular comparison.

Document retrieval from the vector store, triggered by the query, is considered successful if the target protocol appears among the top four retrieved protocols, with errors assessed manually.

Statistical analysis was performed in Python (Version: 3.12.3, [23]) using the additional packages: Scikit-learn, Statsmodels, and SciPy (Versions: 1.5.1, 0.14.3, and 1.14.0, respectively).

## Results

### Study population

After exclusion, 100 reports remained for analysis. The dataset characteristics, including demographic information and the most commonly selected protocols for the patients in the GT, are summarized in Table 1.

**Table 1:** Dataset Characteristics

	Overall
Age(Mean Age + SD)	54.2 + 18.41
Women (in %)	50
Brain protocols	98
Spine protocols	2
Most common protocols	
Tumor-metastases	19
Tumor-Follow-Up	11
Tumor-Early -MRI	9
Inflammation-standard	9
Vascular-infraction/TIA	6
Others	46

SD: Standard Deviation, TIA: Transient Ischemic Attack

### LLM predictions with and without RAG

The accuracies for predicting MRI sequences without RAG were 38% (95% CI: 0.35–0.41) for LLaMA 3.1 405B and 43% (95% CI: 0.4–0.45) for GPT-4o. For contrast medium prediction, LLaMa 3.1 405B achieved an accuracy of 77% (95% CI: 0.69–0.85), while GPT-4o achieved 79% (95% CI: 0.71–0.87). When incorporating RAG, LLaMa 3.1 405B reached an accuracy of 70% (95% CI: 0.65–0.76) for MRI sequence prediction and 94% (95% CI: 0.89–0.98) for contrast medium prediction. GPT-4o with RAG achieved an accuracy of 81% (95% CI: 0.75–0.86) for MRI sequence prediction and 92% (95% CI: 0.86–0.97) for contrast medium prediction.

The implementation of RAG resulted in significantly higher accuracies for sequence prediction and contrast medium administration in both models (Sequence Prediction: GPT-4o 43%, GPT-4o with RAG 81%, *P* < 0.001; LLaMA 3.1 38%, LLaMA 3.1 with RAG 70%, *P* < 0.001, Contrast Medium Prediction: GPT-4o 79%, GPT-4o with RAG 92%, *P* = 0.006, LLaMA 3.1 77%, LLaMA 3.1 with RAG 94%, *P* < 0.001). Comparing both models, GPT-4o yielded significantly higher accuracies in predicting MRI sequences (GPT-4o with RAG 81%, LLaMA 3.1 with RAG 70%, *P* < 0.001). Conversely, in predicting the administration of contrast medium, LLaMA 3.1 showed a marginally superior accuracy, but without reaching statistical significance (GPT-4o with RAG 92%, LLaMA 3.1 with RAG 94%, *P* = 0.48).

**Fig. 4:**
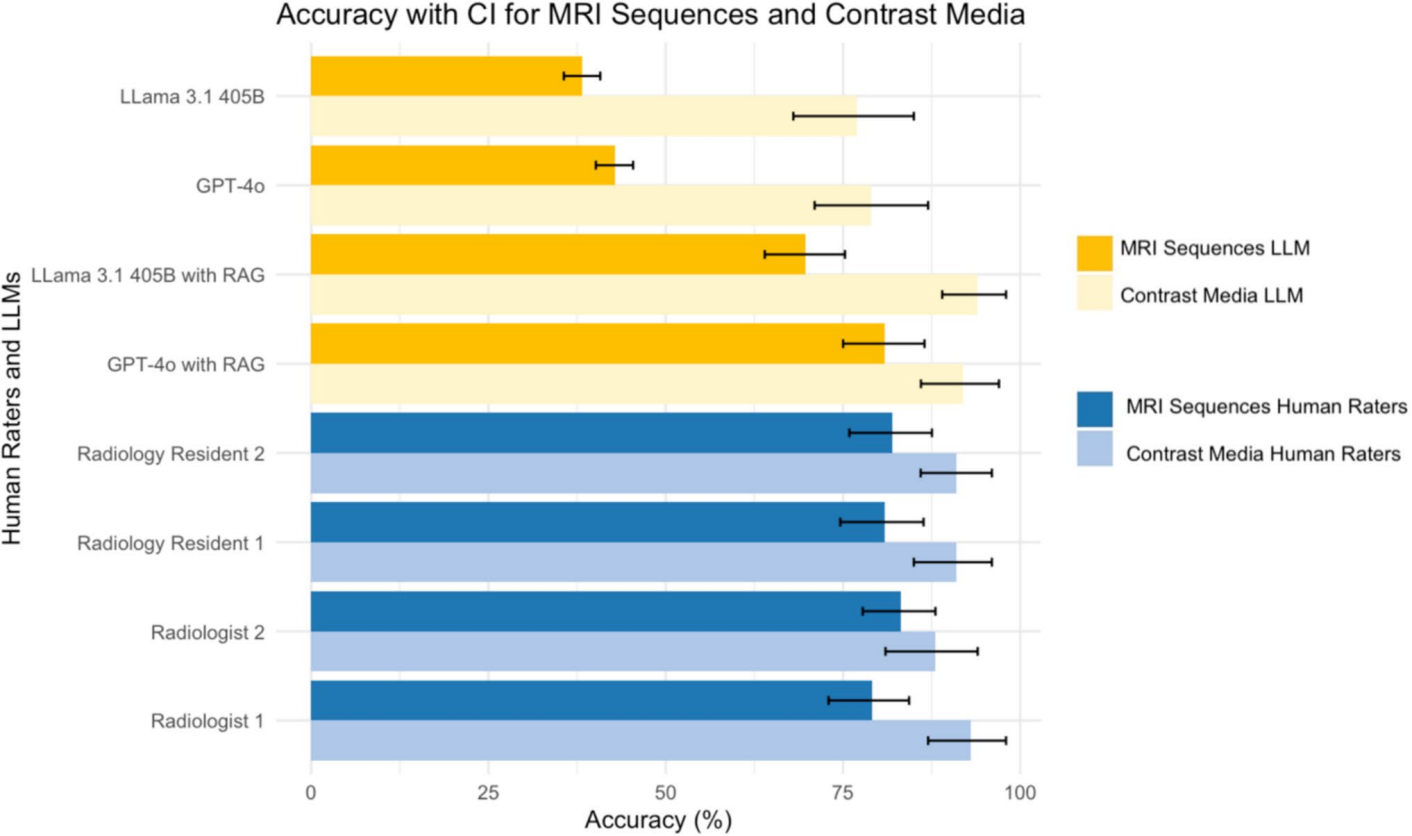
**Accuracies of Protocol Prediction**. Error bars indicate the respective 95% confidence interval (CI). LLM = Large Language Model

Protocol prediction, meaning both predictions for MRI sequences and contrast medium administration, of employed LLMs and tasked radiologists, in comparison to ground truth, is illustrated in Fig. 4.

Table 2 presents translated output examples from both models with RAG, alongside the corresponding GT, for four randomly selected clinical questions.

**Table 2:**
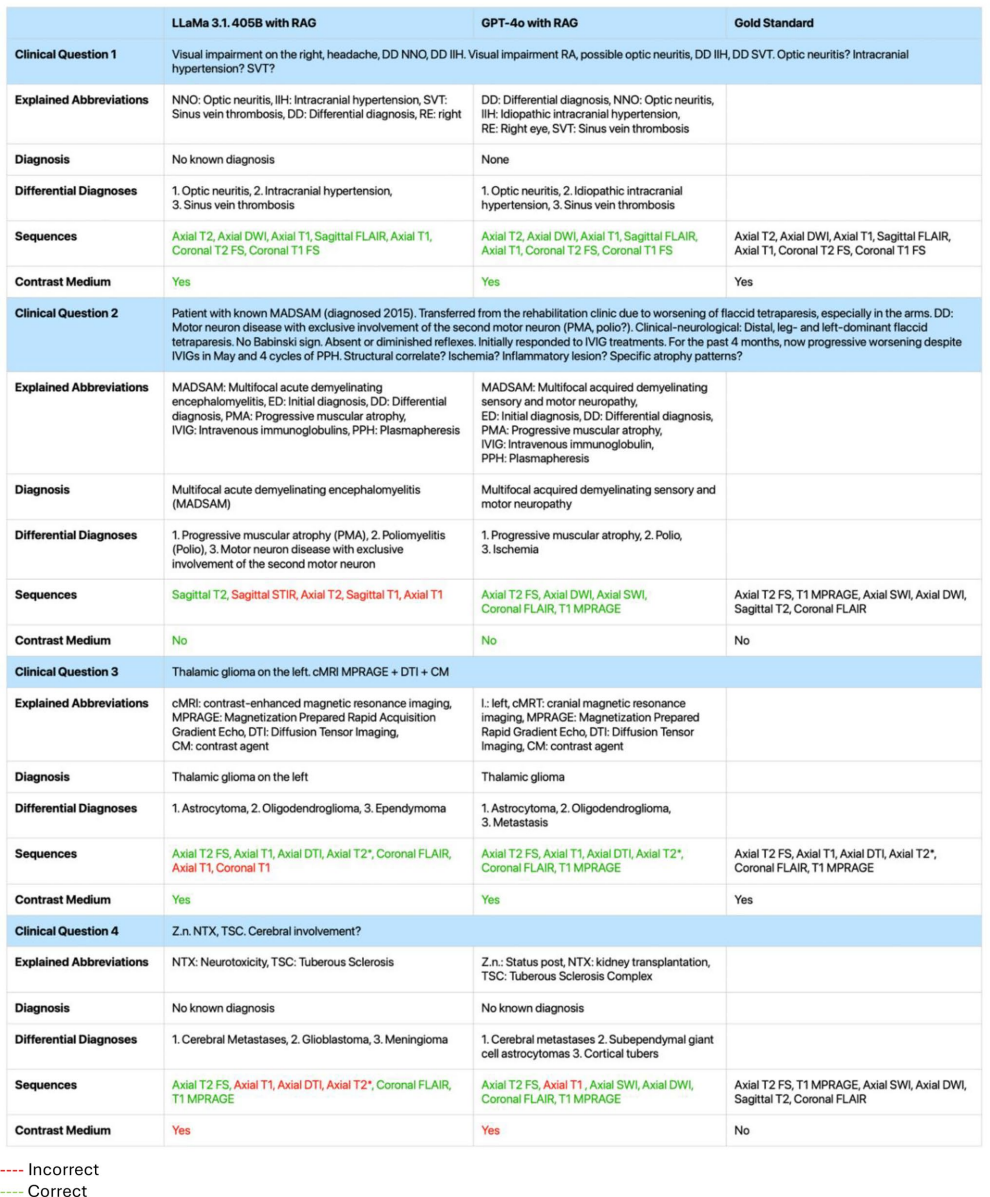
Output Examples of Large Language Models and Gold Standard

Red Incorrect; Green Correct

To ensure transparency, we present examples of generated responses to four clinical questions from both large language models, translated into English. For sequence and contrast medium selection, the Gold Standard answer is provided for comparison. Color coding indicates output accuracy—green for alignment and red for misalignment with the Gold Standard. RAG = Retrieval-Augmented Generation

### Protocol selection of radiologists

In protocol selection, Radiology Resident 2 achieved an accuracy of 82% (95% CI: 0.76–0.88, κ = 0.67) for MRI sequences and 91% (95% CI: 0.85–0.96, κ = 0.78) for contrast medium selection. Radiology Resident 1 achieved an accuracy of 81% (95% CI: 0.75–0.86, κ = 0.59) for MRI sequences and 91% (95% CI: 0.86–0.96, κ = 0.78) for contrast medium. Radiologist 2 selected protocols with an accuracy of 83% (95% CI: 0.78–0.88, κ = 0.61) for MRI sequences and 88% (95% CI: 0.82–0.94, κ = 0.72) for contrast medium. Radiologist 1 had an accuracy of 79% (95% CI: 0.74–0.85, κ = 0.56) for MRI sequences and 93% (95% CI: 0.88–0.97, κ = 0.83) for contrast medium administration. The calculated mean accuracy for all four radiologists was 81% ± 0.21 for MRI sequences and 91% ± 0.24 for contrast medium administration.

When comparing these results to GPT-4o with RAG, no significant differences were found in either task (Sequences: Radiologists 81%, GPT-4o with RAG 81%, *P* = 0.89; Contrast Medium: Radiologists 91%, GPT-4o with RAG 92%, *P* = 0.48). Similarly, radiologists and LLaMA 3.1 had no significant difference in contrast medium prediction (Radiologists 91%, LLaMA 3.1 94%, *P* = 0.48). However, radiologists achieved significantly higher accuracy in sequence prediction (Radiologists 81%, LLaMA 3.1 70%, *P* < 0.001).

### RAG-system

The RAG system correctly retrieved protocols for 81 out of 100 clinical questions using GPT-4o and 78 using LLaMA 3.1. Most retrieval errors were due to the selection of overly specific protocols. In these cases, vague clinical questions required general protocols capable of addressing multiple differential diagnoses, but the system retrieved disease-specific protocols focused on a single symptom or condition. Errors involving confusion between protocols within the same category (e.g., tumor follow-up vs. recurrence) were classified as ‘incorrect protocol within the correct subgroup’. Additional errors included misinterpretation of medical terminology, confusion between brain and spine protocols, and retrieval driven by prior diagnoses rather than the patient’s current symptoms. A detailed breakdown of error types is provided in Table 3.

**Table 3:** Error-Evaluation of Document Retrieval

Type of error in document retrieval	LLaMA 3.1 405B with RAG	GPT-4o with RAG
Overly specific protocols	10	8
Incorrect protocol in the correct subgroup	5	4
Misunderstanding of medical terms	4	4
Incorrect body region	2	2
Protocol retrieval based on pre-diagnosis	1	1

Overly specific protocols = vague clinical question requires a general protocol for broad differential diagnoses, disease-specific protocols are chosen instead; Incorrect protocol in the correct subgroup = confusion between protocols within the same category (e.g., tumor follow-up vs. recurrence); Misunderstanding of medical terms = misinterpretation of medical language; Incorrect body region = brain and spine protocols mistakenly interchanged; Protocol retrieval based on pre-diagnosis = focus on past diagnosis rather than current symptoms; RAG = Retrieval-Augmented Generation

## Discussion

The automation of radiology protocol assignment holds significant potential to reduce the daily workload of radiologists while maintaining safety and high quality care. In this study, we evaluated the capabilities of retrieval-augmented open-source and proprietary LLMs to predict MRI protocol assignments in neuroradiological practice.

The use of RAG led to a significant increase in sequence prediction accuracy for LLaMa 3.1 405B from 38 to 70% (*P* < 0.001) and for GPT-4o from 43 to 81% (P < 0.001), achieving at least a 1.8-fold improvement compared to performance without RAG. OpenAI’s GPT-4o demonstrated significantly superior accuracy compared to the open-source model for MRI sequence prediction (*P* < 0.001) achieving levels comparable to those of radiologists (*P* = 0.89).

Several studies have explored automating MRI protocol selection using machine learning [4–9]. Research incorporating protocols across all body regions or focusing solely on musculoskeletal imaging achieved similar accuracies (83–87%) using convolutional neural networks or support vector machines [4–7]. In neuroradiology, Brown et al. identified gradient boosting machines as the most effective model with 95% accuracy, while Chillakuru et al. employed natural language processing (fastText, XGBoost), achieving 83% accuracy for spine and 85% for brain MRI. However, task complexity varies significantly across studies. While we incorporated the full neuroradiological set of 63 protocols, others included solely two to nine protocols, which does not reflect the complexity in clinical routine [4, 8]. Importantly, our approach preserved the original clinical question format encountered by radiologists, leveraging the free-text processing capabilities of LLMs. In contrast, previous studies required preprocessing to accommodate free-text input with machine learning models. However, free-text input of clinical indication is crucial, since protocol selection cannot rely solely on admitting diagnoses or extracted features, as they lack sufficient clinical detail [28]. Despite these complexities, GPT-4o's sequence selection accuracy, when combined with RAG, reached 81%, which is comparable to the results of Chillakuru et al. While their errors tended to select overly general protocols, our model’s errors were primarily due to overly specific choices. The key limitation across all of these studies is the challenge machine learning models face in generalizing due to non-standardized MRI sequence nomenclature and institution-specific protocols. Frequent equipment updates and evolving protocol guidelines further necessitate periodic model retraining—a process that requires expert input, time, and training-associated costs, making it difficult for these models to adapt seamlessly to new information.

Gertz et al. investigated GPT-4’s ability to predict the appropriate imaging modality based on clinical questions, achieving an overall accuracy of 84% in identifying imaging modality (X-ray, scintigraphy, CT, MRI), anatomical region, and contrast phase [16]. However, their model did not address specific MRI protocols or sequences in detail. Our study advances this work by automating MRI protocol assignment across a wide range of institution-specific protocols, achieving high accuracy in retrieving detailed protocol information.

Kim et al. investigated the automation of MRI protocol assignment in neuroradiology using LLMs alone. In a study design similar to ours, both open-source and proprietary LLMs were tasked with assigning MRI protocols, with and without access to external medical knowledge. However, instead of using RAG, their study employed in-context learning by supplying institutional MRI protocols and corresponding explanations directly within the prompt. Overall, OpenAI’s o3-mini achieved the highest performance, followed by GPT-4o and the open-access models DeepSeek-R1 and Qwen-2.5-72B. In line with our findings, incorporating external knowledge enhanced the accuracy of all evaluated models. In-context learning emerges as an alternative to RAG, as the context windows of LLMs continue to expand [10]. However, this approach substantially increases the token count, resulting in longer inference times, higher computational costs and energy consumption. Moreover, RAG offers greater transparency by enabling objective traceability of the source documents used in generating the LLM’s response. This helps mitigate the commonly cited 'black box' limitation of LLMs [29]. Given the importance of time-efficiency and source traceability in clinical settings, RAG-based approaches demonstrate greater practical applicability in healthcare than LLMs alone.

RAG, as an innovative approach, is rapidly advancing in the medical field [29], demonstrating high accuracy in various tasks, such as disease-specific treatment recommendations [18, 21], guideline-based clinical decision support [19, 20], and radiology-specific queries [22]. For instance, Ferber et al. demonstrated RAG’s effectiveness in oncology treatment decision-making, observing an accuracy increase from 57 to 84% when implemented with GPT-4. This underscores RAG’s potential for significantly enhancing LLM-powered decision-support systems in complex clinical settings. Simultaneously, RAG remains an active area of research, with ongoing advances in architectures aimed at improving retrieval accuracy and overall model performance. Agentic RAG introduces multi-step, iterative use of LLMs to enhance planning, reflection, and reasoning [30], while hybrid search improves retrieval by combining semantic and keyword-based search [31]. Both developments hold particular promise for optimizing RAG applications in healthcare.

Unlike prior studies comparing open-source and proprietary models, LLaMa 3.1 405B, the open-source model used in this study, performed significantly worse on the main task [32, 33]. Previous research has often confined LLMs to multiple-choice questions within structured frameworks, while our study used a more open-ended approach, increasing the complexity of the decision-making process. This suggests that as task complexity increases, the performance gap between open-source and proprietary models may widen. However, proprietary models cannot be deployed locally, which presents a notable limitation: data privacy challenges restrict their clinical applicability, as patient data requires thorough de-identification—a complex process—prior to use [33]. Encouragingly, advancements in data privacy or improvements of open-source LLMs may soon address these limitations.

Data privacy is not the only challenge that must be addressed for the clinical application of LLM-based tools. Hallucination remains a significant concern [29], although strategies such as RAG have been shown to mitigate this issue and demonstrably reduce its occurrence. Prediction errors in protocol assignment can result in critical findings being missed during MRI examinations, potentially necessitating patient recall—an outcome that is both time-consuming, and in the worst case, detrimental to patient health [34]. Consequently, AI-based tools used in clinical settings are classified as medical devices and are subject to strict regulatory oversight. A fully autonomous system for radiologists’ decision-making in protocol selection is, therefore, currently unattainable. However, a further refined LLM- and RAG-powered tool, operating under continued human supervision—similar to the proposed integration of the NLP model [8]—could offer meaningful support for radiologists.

Our study has its limitations. First, the retrieval of relevant documents did not consistently function as intended, which in some cases prevented the LLM from selecting the correct protocol. During the study, we addressed this issue through prompt engineering and by increasing the number of retrieved documents, which led to substantial improvements. However, further optimization is necessary to fully leverage the potential of LLMs—for example, by implementing advanced RAG architectures or enhancing retrieval through improved search techniques such as hybrid search, rather than relying solely on semantic search [30, 31]. Second, protocol selection varies, as radiologists may use different but valid approaches to achieve the same diagnostic goals. For example, a radiologist might choose Protocol 1 and add sequences from Protocol 2, an option not allowed in our study, but could be overcome by instructing the LLM to suggest multiple protocols. Third, the models lacked time stamps, and clinical questions sometimes mentioned symptom onset without specific intervals, complicating decisions for time-sensitive protocols, such as stroke protocols in acute or subacute phases. Future studies should integrate temporal data. Fourth, our results are based on the model’s performance in protocol assignment in German language, and therefore may not be directly applicable in other languages. However, previous studies have shown that LLMs achieve comparable task performance in English and German [35]. Lastly, the small sample size may limit the generalizability of our findings.

## Conclusion

MRI protocol assignment is a routine yet time-consuming and error-prone task in radiology, highlighting the clinical need for automation. We demonstrated that automated MRI protocoling, using a pipeline that integrates a large language model with retrieval-augmented generation, achieves high accuracy in predicting institution-specific protocols. This approach represents a novel solution for automated protocol assignment, distinct from previous studies with strong potential for long-term, cross-institutional implementation. Given the rapid advancements in computer science and artificial intelligence, future research should focus on adapting the model to emerging technologies, particularly by optimizing the retrieval process. Additionally, investigating the model’s transferability to other imaging modalities and anatomical regions would be of significant clinical value.

## Data Availability

The source code for the methodology used in this study is available on GitHub (https://github.com/laranoellereiner/LLM_RAG_Neuroradiology). The research data are not publicly available. However, inquiries regarding the analyzed data can be directed to the corresponding author.

## Abbreviations

AI: Artificial intelligence
GT: Ground Truth
LLM: Large language model
RAG: Retrieval-augmented generation
